# “Clinical Overlap of Darier's Disease and Acrokeratosis Verruciformis of Hopf”: A Case Report

**DOI:** 10.1002/ccr3.70862

**Published:** 2025-09-08

**Authors:** Mahesh Mathur, Sumit Paudel, Sandhya Regmi, Sambidha Karki, Shilpa Maharjan, Nabita Bhattarai

**Affiliations:** ^1^ Department of Dermatology College of Medical Sciences Bharatpur Nepal

**Keywords:** Acrokeratosis Verruciformis of Hopf, *ATP2A2 gene*, *clinical overlap*, Darier's diease, mutation

## Abstract

Darier's disease and Acrokeratosis Verruciformis of Hopf can exhibit overlapping clinical features due to mutations in the same ATP2A2 gene. Recognizing this genetic and phenotypic overlap is crucial for accurate diagnosis, genetic counseling, and treatment, especially in mixed presentations of these rare genodermatoses.

## Graphical abstract

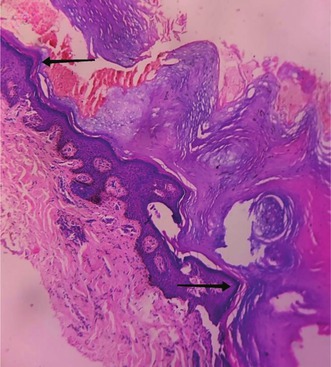

**Abbreviations**
AKVH: Acrokeratosis Verruciformis of Hopf
DD: Darier's disease
HPE: Histopathological examination
SERCA2: Sarcoendoplasmic reticulum Ca2+ ATPase 2

## 1 Introduction

Acrokeratosis Verruciformis of Hopf (AKVH) and Darier's Disease (DD) are rare autosomal dominant genodermatoses but may occur sporadically. AKVH develops in early childhood or may have late onset in the fifth decade of life with no gender predilection. The disease clinically presents as multiple skin‐colored, plane wart‐like lesions, primarily on the dorsum of the hands and feet [1, 2].

DD develops at puberty and is clinically characterized by yellowish brown, greasy, and crusted papules or plaques in seborrheic areas of the body, nails, and oral mucosa. Both genodermatoses result from a mutation in the ATP2A2 gene on chromosome 12q24 [3]. AKVH and DD are considered as allelic disorders [4].

Histopathological examination (HPE) of skin biopsy is the gold standard in the diagnosis of both AKVH and DD [4]. Hereby, we have highlighted the clinical overlap of the two diseases in a patient, confirmed by HPE in the absence of ATP2A2 sequencing.

## 2 Case History and Examination

**FIGURE 1:**
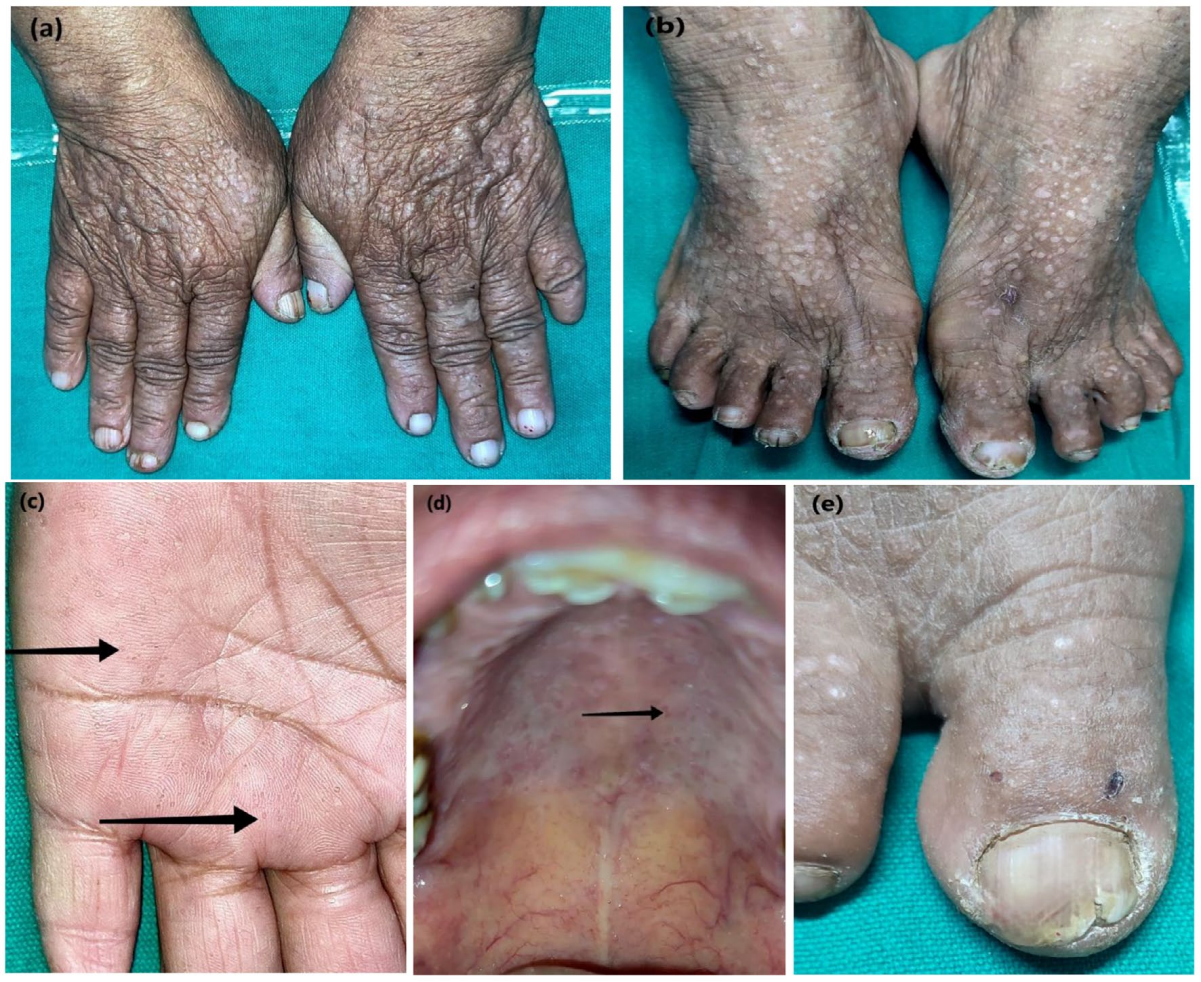
Acrokeratosis Verruciformis of Hopf and Darier's disease: (a) Multiple, discrete, firm, flat topped skin colored papules on bilateral dorsum of hand, (b) on dorsum of bilateral foot, (c) Multiple pits in palms, (d) Pits in hard palate, (e) “V” shaped nick at free margin of toe nail.

A 58‐year‐old female presented with asymptomatic, multiple, discrete, firm, flat‐topped, and skin‐colored papules for the last 5 years. The lesion first appeared on the face which gradually progressed to involve the dorsum of the hands and feet bilaterally (Figure 1a,b). There were multiple pits observed over the bilateral palms and hard palate (Figure 1c,d); V‐shaped nicks and splits at the free margins of the nail plate (Figure 1e). There was no history of similar illness in other family members.

## 3 Differential Diagnosis

Plane wart, DD, AKVH and epidermodysplasia verruciformis were initially considered in the differential diagnosis.

## 4 Methods

**FIGURE 2:**
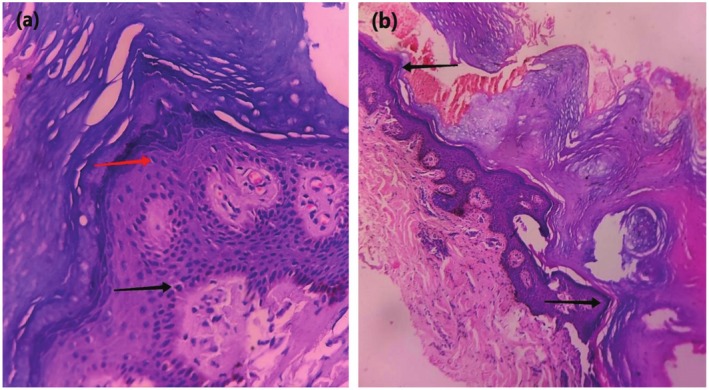
Histopathology of Darier's disease and Acrokeratosis Verruciformis of Hopf: (a) Hematoxylin and eosin (H/E stain at 40×) Hyperkeratosis, acantholysis, suprabasal clefting, few corps ronds (black arrow) and grains (red arrow) typical of DD; (b) Hematoxylin and eosin (H/E stain at 10×) Hyperkeratosis, acanthosis, hypergranulosis, and papillomatosis resembling “church spires” (black arrow) typical of AKVH.

Routine baseline investigations, except triglycerides, were within normal limits. HPE of skin biopsy taken from the left forehead revealed hyperkeratosis, acantholysis, supra‐basal clefting, and few corps ronds, typical of DD (Figure 2a), while HPE of skin biopsy taken from the dorsum of the right foot showed hyperkeratosis, acanthosis, hypergranulosis, and papillomatosis resembling “church spires” typical of AKVH (Figure 2b).

## 5 Treatment

Based on the clinical and histopathological examinations, the patient was found to have overlap features of both DD and AKVH. The patient had a deranged lipid profile, so oral retinoids were avoided. She was started on topical retinoids (tretinoin 0.05%) daily at night time and advised to follow‐up in the next 6 weeks.

## 6 Outcome and Follow‐Up

Minimal improvement in lesions was noticed in the first follow‐up visit. The patient was counseled for other treatment options such as cryotherapy and laser. However, being aware of the prognosis of the disease and treatment expenses, the patient denied all other treatment options and later lost follow‐up.

## 7 Discussion

DD and AKVH are inherited as an autosomal dominant trait but can occur sporadically, as in our case. Pathogenesis in both of these conditions involves a missense mutation in the *ATP2A2 gene*, *which encodes* sarcoendoplasmic reticulum Ca^2+^ ATPase 2 (SERCA2), resulting in impaired Ca^2+^ transport across the cell membrane, dysregulated intracellular calcium signaling, impaired cell–cell adhesion, and dyskeratosis [3]. Mutation in the same gene, that is, the ATP2A2 gene, explains the overlapping clinical features of DD and AKVH observed in our patient. This highlights the concept of the same genetic etiology with a different phenotypic spectrum rather than two separate disorders.

DD has a predilection for sebaceous areas, typically involves oral mucosa and nails, whereas AKVH does not affect sebaceous areas; nail findings and oral mucosal involvement are rare [5]. However, punctate pits in palms and soles are present commonly in AKVH and rarely in DD [4].

Classic histopathological features of DD include hyperkeratosis, parakeratosis, acantholysis, supra‐basal cleft, corps ronds, and grains, while that of AKVH includes hyperkeratosis without parakeratosis, acanthosis, circumscribed epidermal elevations or papillomatosis, resembling church spires within the stratum corneum [1, 4]. We also reported similar histopathological findings from two different lesions in our case.

Harman et al. [6] reported cytological examination of AKVH lesions showing dyskeratotic acantholytic cells, corps ronds, and grains, similar to DD. He also demonstrated certain histopathological features in lesions of AKVH overlapping with those of DD. Similarly, Bergman et al. [7] also demonstrated overlapping histopathological features of AKVH lesions with DD. However, we did not find such overlapping histopathological features in our patient.

Clinical presence of papular lesions in the forehead and infraorbital region, multiple pits in the hard palate, “v” shaped nicks at the free margin of nails and longitudinal ridgings, and supra‐basal splitting and corps ronds in histopathological examination confirmed the diagnosis of DD, whereas multiple, warty, flat topped papules in the dorsum of hands and feet, bilateral palmar pits with typical “church spires” in histopathology confirmed the diagnosis of AKVH in our case (Table 1).

**TABLE 1:** Comparative clinical and histopathological features of Darier's disease and Acrokeratosis Verruciformis of Hopf.

Features	Darier's disease	Acrokeratosis verruciformis of hopf
Cutaneous features	Yellowish, greasy and crusted papules or plaques in seborrheic areas of the body Bilateral palmar pits rarely seen	Multiple skin‐colored, wart‐like lesions, primarily on dorsum of hands and feet Bilateral palmar pits frequent and is a characteristic finding
Nail changes	V‐shaped nick at distal free margin (common, often diagnostic)	Rare
Oral mucosa	Commonly involved	Rare
Histopathological features		
Acantholysis	Present	Absent
Dyskeratosis	Corps ronds, grains present	Absent
Hyperkeratosis	Present	Present
Papillomatosis	Mild	Characteristic “church spire” pattern

Piskin et al. [4] reported the coexistence of DD and AKVH in 31‐year‐old woman with a similar history in other family members. Palmar and mucosal lesions were not observed in this case report. In our patient, we observed similar clinical and histological findings. However, the disease onset in our case was quite late, that is, in her fifties, with no involvement of other family members. We also reported palmar as well as mucosal lesions in our case, which have been mentioned very rarely in the literature so far.

Likewise, Dhitavat et al. [8] reported loss of function of the sarcoplasmic reticulum Ca^2+^ ATPase 2 mutant in AKVH, providing evidence that AKVH and DD are allelic disorders. Matsumoto et al. [9] reported detection of human papillomavirus‐17 DNA in the verruca plana–like papule of AKVH and Tummidi et al. [10] recently reported a nonfamilial case of AKVH.

Conglomeration of typical clinical findings and histopathology of skin biopsy confirmed features of both DD and AKVH in our patient, which is a rare finding and only a few cases have been reported in the literature. Mutational analysis is needed to identify the specific mutation involved in both diseases. However, we were unable to perform ATP2A2 sequencing due to financial inadequacies.

The clinical findings of AKVH may be easily confused with DD, epidermodysplasia verruciformis, seborrheic keratosis, and plane wart. Histopathological examination is usually required to differentiate these conditions [6]. Superficial ablation is considered to be the most effective treatment modality. Other treatment options are keratolytic solutions, topical retinoids, acitretin, cryotherapy, laser therapy, and surgical excision [5].

Our case demonstrated clinical overlap between DD and AKVH revealing phenotypic variations within a single genodermatosis spectrum. An updated literature to date has failed to show an overlap in mutations in AKVH and DD. Transformation of AKVH to squamous cell carcinoma has been reported in the literature. Therefore, timely monitoring of skin lesions is important to identify the risk of developing squamous cell carcinoma in these patients.

## Author Contributions

**Mahesh Mathur:** conceptualization, methodology, supervision, validation. **Sumit Paudel:** data curation, formal analysis, resources, validation. **Sandhya Regmi:** formal analysis, software, supervision, writing – review and editing. **Sambidha Karki:** conceptualization, data curation, supervision, visualization. **Shilpa Maharjan:** data curation, methodology, supervision, writing – review and editing. **Nabita Bhattarai:** conceptualization, methodology, resources, writing – original draft.

## Disclosure

Prior publication: This material has not been published previously.

## Ethics Statement

Reviewed and approved by Institutional Review Board College of Medical Sciences (IRBCOMS).

## Consent

The authors obtained written consent from the patient for use of photographs and medical information to be published online and with the understanding that this information may be publicly available and discoverable via search engines. Patient consent forms are not provided to the journal but are retained by the authors.

## Conflicts of Interest

The authors declare no conflicts of interest.

## Data Availability

The data that support the findings of this study are available from the corresponding author upon reasonable request.
